# Adalimumab biosimilar uptake among dermatologists in Medicare Part D through 2023: A retrospective analysis using the Prescribers – by Provider and Drug dataset

**DOI:** 10.1016/j.jdin.2026.07.006

**Published:** 2026-07-28

**Authors:** Nathan C. Schedler, Manaal Saqib, Chenan A. Huang, Steven R. Feldman

**Affiliations:** a Wake Forest University School of Medicine, Winston-Salem, North Carolina; b Department of Dermatology, Center for Dermatology Research, Wake Forest University School of Medicine, Winston-Salem, North Carolina; c Department of Dermatology, Wake Forest University School of Medicine, Winston-Salem, North Carolina; d Department of Pathology, Wake Forest University School of Medicine, Winston-Salem, North Carolina; e Department of Social Sciences & Health Policy, Wake Forest University School of Medicine, Winston-Salem, North Carolina

**Keywords:** adalimumab prescription, biosimilar prescription, dermatology, Medicare Part D

*To the Editor:* Adalimumab was revolutionary for inflammatory skin disease but is costly.^1^^,^^2^ Adalimumab biosimilars first received FDA approval in 2016, but market entry was delayed until 2023 due to patent litigation and exclusivity agreements.^3^ Our objective was to quantify real-world uptake of adalimumab biosimilars in the first year following market entry and identify specialty-specific adoption patterns, providing early suggestion on whether biosimilar are achieving their intended goal of expanding biologic access.

CMS Medicare Part D data were filtered to assess total adalimumab claims, number of prescribing dermatologists, and claims per dermatologist for originator and biosimilar adalimumab by year and state.^4^ National totals and rates were compared across other high-prescribing specialties. Testing included z-tests for biosimilar adoption across specialties, *t*-tests comparing claims per dermatologist across years, and ANOVA tests comparing state-level totals to identify potential geographic patterns or clusters in biosimilar adoption. No correction for multiple hypothesis testing was applied due to the exploratory nature of the analysis.

In 2023, there were 50,463 total adalimumab claims in dermatology, consisting of 1728 (3.42%) biosimilar and 48,735 (96.58%) originator adalimumab. Biosimilar adoption in dermatology was low and similar to rheumatology, gastroenterology, and ophthalmology (*P* > .05, Table I). State-level analysis revealed no significant geographic variation, suggesting uniform low initial adoption nationally. Between 2020 and 2022, adalimumab claims and prescribing dermatologists increased year-to-year. In 2023, with biosimilar introduction, differences were small and unlikely to be clinically meaningful (Table II).

**Table I:** Utilization and market share of biosimilar adalimumab in dermatology, rheumatology, gastroenterology, and ophthalmology after the first year of launch (2023)

Specialty	Total adalimumab claims	Biosimilar adalimumab claims	Biosimilar adalimumab share (%)
Dermatology	50,463	1728	3.42%
Rheumatology	284,238	6137	2.16%
Gastroenterology	58,672	947	1.61%
Ophthalmology	3076	66	2.15%

**Table II:** Annual volume and prescriber rate of originator and biosimilar adalimumab claims among U.S. dermatologists (2020-2023)

Year	Total adalimumab claims (originator + biosimilar)	Number of dermatology prescribers	Average claims per dermatology prescriber
2020	45,369	2004	22.64
2021	47,658	2082	22.89
2022	50,966	2217	22.99
2023	50,463	2220	22.73

In the calendar year 2023, which included the initial staggered launch of biosimilars, adoption was limited. Biosimilars are FDA-approved based on pharmacokinetic similarity and extrapolation from rheumatology and gastroenterology trials, without dermatology-specific efficacy studies. While regulatory agencies deemed this sufficient for interchangeability designation, the lack of dermatology trial data may contribute to prescriber uncertainty. However, low utilization may not represent clinician intent but instead reflect insurance formularies, pharmacy benefit manager decisions, prior authorization requirements, and automatic substitution policies. Our prescriber-level data cannot distinguish between active prescriber choice and payer-mandated restrictions. The FDA's recent granting of interchangeability status to certain adalimumab biosimilars may accelerate future adoption by allowing easier pharmacy-level substitution.

This analysis has limitations. First, Medicare Part D predominately reflects an older population and may not generalize to younger patients or those with commercial insurance. Second, our dataset captured claims volume but not actual transaction prices or rebates, so we could not directly quantify cost savings. Third, these results represent only the first year of market availability; adoption may increase as logistical barriers resolve requiring the need for longitudinal evaluation. Additionally, prescribing patterns show that while biosimilar entered the market in 2023, newer biologics (IL-17 and IL-23 inhibitors) may be preferentially used for conditions where adalimumab was historically an option, potentially limiting long-term biosimilar impact (Supplementary I, available via Mendeley at https://data.mendeley.com/datasets/7fvy768mjv/1).

Dermatologists have limited control over formulary decisions but play a key role in patient education and navigating insurance requirements. Quantifying early adoption patterns provides essential evidence for policymakers, designing interventions to accelerate biosimilar integration. Continued evaluation is necessary to determine if biosimilars successfully meet goals of enhanced affordability and access.

### Declaration of generative AI and AI-assisted technologies in the writing process

AI was not used in the manuscript composition.

## Conflicts of interest

Dr Feldman has received research, speaking, and/or consulting support from Eli Lilly and Company, GlaxoSmithKline/Stiefel, AbbVie, Janssen, Alovtech, vTv Therapeutics, Bristol-Myers Squibb, Samsung, Pfizer, Alumis, Boehringer Ingelheim, Oruka, Amgen, Dermavant, Arcutis, Novartis, UCB, Helsinn, Sun Pharma, Almirall, Galderma, Leo Pharma, Mylan, Celgene, Ortho Dermatology, Menlo, Merck & Co, Qurient, Forte, Arena, Biocon, Accordant, Argenx, Sanofi, Regeneron, the National Biological Corporation, Caremark, Teladoc, BMS, Ono, Micreos, Eurofins, Informa, UpToDate, Verrica, and the National Psoriasis Foundation. He is the founder and part owner of Causa Research and holds stock in Sensal Health. Authors Schedler, Saqib, and Dr Huang have no conflicts of interest to declare.
